# MiR-206 inhibits HGF-induced epithelial-mesenchymal transition and angiogenesis in non-small cell lung cancer via c-Met /PI3k/Akt/mTOR pathway

**DOI:** 10.18632/oncotarget.7570

**Published:** 2016-02-22

**Authors:** Qing-yong Chen, De-min Jiao, Yu-quan Wu, Jun Chen, Jian Wang, Xia-li Tang, Hao Mou, Hui-zhen Hu, Jia Song, Jie Yan, Li-jun Wu, Jianyan Chen, Zhiwei Wang

**Affiliations:** ^1^ Department of Respiratory Disease, The 117^th^ Hospital of PLA, Zhejiang, China; ^2^ Department of Oncology, The 117^th^ Hospital of PLA, Zhejiang, China; ^3^ Department of Anesthesiology, Shenzhen Baoan Hospital Affiliated to Southern Medical University, Guangdong, China; ^4^ Department of Pathology, Beth Israel Deaconess Medical Center, Harvard Medical School, Boston, MA, USA; ^5^ The Cyrus Tang Hematology Center and Collaborative Innovation Center of Hematology, Jiangsu Institute of Hematology, The First Affiliated Hospital, Soochow University, Suzhou, China

**Keywords:** miR-206, HGF, epithelial-mesenchymal transition, angiogenesis, lung cancer

## Abstract

MiR-206 is low expression in lung cancers and associated with cancer metastasis. However, the roles of miR-206 in epithelial-mesenchymal transition (EMT) and angiogenesis in lung cancer remain unknown. In this study, we find that hepatocyte growth factor (HGF) induces EMT, invasion and migration in A549 and 95D lung cancer cells, and these processes could be markedly inhibited by miR-206 overexpression. Moreover, we demonstrate that miR-206 directly targets c-Met and inhibits its downstream PI3k/Akt/mTOR signaling pathway. In contrast, miR-206 inhibitors promote the expression of c-Met and activate the PI3k/Akt/mTOR signaling, and this effect could be attenuated by the PI3K inhibitor. Moreover, c-Met overexpression assay further confirms the significant inhibitory effect of miR-206 on HGF-induced EMT, cell migration and invasion. Notably, we also find that miR-206 effectively inhibits HGF-induced tube formation and migration of human umbilical vein endothelial cells (HUVECs), and the mechanism is also related to inhibition of PI3k/Akt/mTOR signaling. Finally, we reveal the inhibitory effect of miR-206 on EMT and angiogenesis in xenograft tumor mice model. Taken together, miR-206 inhibits HGF-induced EMT and angiogenesis in lung cancer by suppressing c-Met/PI3k/Akt/mTOR signaling. Therefore, miR-206 might be a potential target for the therapeutic strategy against EMT and angiogenesis of lung cancer.

## INTRODUCTION

Lung cancer, the leading cause of cancer deaths, has the most rapidly increasing incidence rate worldwide [1]. Non-small-cell lung cancer (NSCLC) is the most common form of lung cancer. About 40% of patients with NSCLC present at an advanced stage, with metastatic or locally advanced disease, and nearly 90% of lung cancer patients die of metastasis [2]. Therefore, novel therapeutic approaches to block tumor invasion and metastasis are urgently needed.

Epithelial to mesenchymal transition (EMT) is a vital process in the conversion of early-stage tumors into invasive malignancies. It has been shown that the EMT is associated with cancer development and metastasis [3]. Cancer cells with EMT phenotype change often involve in epithelial characteristics loss and mesenchymal properties acquisition, exhibiting enhanced motility and invasive abilities [4]. A typical characteristic of the EMT process is the mesenchymal markers such as fibronectin, vimentin and N-cadherin increased, while epithelial markers decreased like E-cadherin, which induces disruption of cell-to-cell junctions. Angiogenesis also plays a major role in tumor growth, progression, and metastasis. As tumors progress, nutrients and oxygen become depleted within the core which induces the production of angiogenic growth factors [5]. These growth factors bind to receptors on nearby quiescent endothelial cells (EC) in pre-existing capillaries, leads to their activation, proliferation and ultimately formation of new vessels [6].

EMT and angiogenesis can be induced by various growth factors, including vascular endothelial growth factor (VEGF), fibroblast growth factor (FGF), platelet-derived growth factor (PDGF) and hepatocyte growth factor (HGF) [7]. Among them, HGF (also known as scattering factor) activates the c-Met signaling pathway, thereby, increasing the invasive and metastatic potentials of the cells and allowing the survival of cancer cells in the bloodstream in the absence of anchorage [8, 9]. The clinical importance of HGF and its receptor c-Met has been further demonstrated in recent studies, showing that the levels of c-Met in mammary cancer tissues and levels of circulating HGF in patients with mammary cancer are associated with a lower survival and development of distant metastasis [10–12]. In addition, HGF is well known as a potent angiogenic cytokine, and c-Met signal activation can modify the microenvironment to facilitate cancer progression [4, 8].

MicroRNAs (miRNAs) are small noncoding RNAs that function as endogenous silencers of numerous target genes. MiRNAs have shown promises in both basic research and clinical application. Hundreds of human miRNAs have been identified in the human genome and some of them are crucially involved in cancer initiation and progression [13, 14]. For example, miR-93 activated c-Met/PI3K (phosphoinosmde-3-kinase)/Akt pathway activity via inhibition of PTEN (phosphatase and tensin homolog deleted on chromosome ten) and CDKN1A (cyclin-dependent kinase inhibitor 1A) in hepatocellular carcinoma [15]. MiR-206 has been well studied in various tumor cells and down-regulated miR-206 is observed in different types of cancers including lung cancer [16–23]. For example, it has been reported that miR-206 plays an important role in breast cancer cell migration and invasion by targeting Cdc42 [24]. MiR-206 can inhibit the expression of VEGF and regulate the apoptosis and migration of laryngeal cancer cells [25]. Decreased expression of miR-206 in breast, malignant astrocytomas and gastric cancer is also associated with disease characteristics and patient survival [16, 19, 22]. MiR-206 overexpression promotes apoptosis, induces cell cycle arrest and inhibits the migration of human hepatocellular carcinoma cells [26]. MiR-206-based therapy is beneficial in cancers where cyclin D1 is overexpressed [27]. In addition, miR-206 is also associated with invasion and metastasis of lung cancer cells [22]. Although multiple anticancer functions of miR-206 have been described, the roles of miR-206 in EMT and angiogenesis of lung cancer remain largely unknown.

In the current study, we demonstrated that miR-206 overexpression inhibited HGF-induced lung cancer EMT, metastasis and angiogenesis by targeting c-Met and its downstream PI3k/Akt/mTOR (mammalian target of rapamycin) pathway. Furthermore, miR-206 showed a significant inhibitory effect on lung cancer growth, EMT and angiogenesis in mouse xenograft tumor model, suggesting the potential use of miR-206 in the targeted therapy of lung cancer.

## RESULTS

### MiR-206 is low expression in lung cancer tissues and cells with high-metastasis potential and correlated with aggressiveness of lung cancer

**Figure 1:**
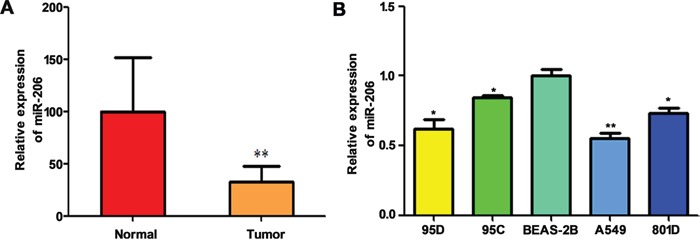
Relative expression of miR-206 in high metastatic NSCLC tissues and cells qRT-PCR showed that miR-206 was downregulated in high metastatic lung cancer tissues (** P<0.01 vs normal) **A.** and lung cancer cell lines **B.** Triplicate assays were performed for each RNA sample, and the relative amount of miR-206 was normalized to U6 snRNA. * *P*<0.05, ** *P*<0.01 vs BEAS-2B group.

Down-regulated miR-206 has been observed in different types of cancers including lung cancer [17, 19]. In this study, we detected the expression levels of miR-206 in 35 NSCLC specimens with lymph node metastasis and 35 para-tumorous tissues by quantitative real-time PCR. The results were shown in Figure 1A, miR-206 was downregulated in cancer tissues compared with the corresponding non-tumor lung tissues. Furthermore, we analyzed the expression of miR-206 in the different human lung cancer cell lines. As shown in Figure 1B, the expression of miR-206 was significantly lower in lung cancer cell lines than in human normal bronchial epithelial cell line, BEAS-2B. Moreover, miR-206 was expressed at significantly lower levels in 95D cells (with relatively high metastatic ability) than in 95C cells (with relatively low metastatic ability), suggested that miR-206 might be involved in the lung cancer metastasis. In the following study, we selected A549 and 95D because these two cell lines have lowest and higher expression of miR-206, respectively.

### HGF induces EMT, migration and invasion of lung cancer cells

**Figure 2:**
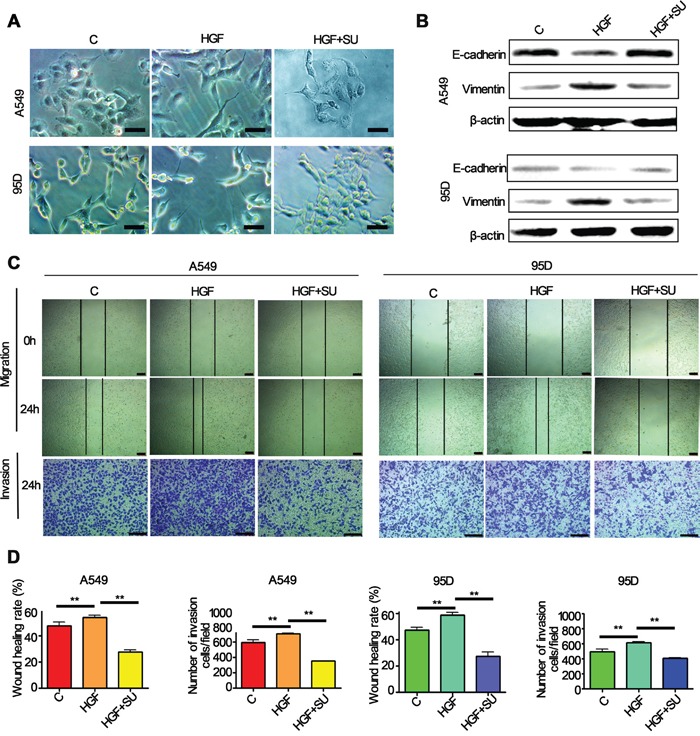
HGF induced significantly EMT translation in lung cancer cells A549 and 95D cells pretreated with or without 5μM c-Met inhibitors, SU11274, for 1h and then stimulated with 50 ng/mL of HGF for 72 hours. **A.** The change in morphology was observed under a light microscope. Both these two cells showed mesenchymal features after the HGF treatment, but pretreatment with SU11274 inhibited HGF induced phenomenon of EMT in A549 and 95D cells. Bar = 50μm. **B.** Western blot analysis showed that E-cadherin expression reduced, and vimentin expression increased in the two cell types after the HGF stimulation. In contrast, SU11274 inhibited the HGF induced vimentin upregulation and E-cadherin downregulation. **C, D.** Wound healing and transwell invasion assays showed that HGF induced migration and invasion of A549 and 95D cells, and the effects can be attenuated by SU11274 pretreatment. Bar = 200μm. Triplicate assays were performed. C: control; SU: SU11274. ** *P*<0.01 vs control.

Previous study has reported that HGF induces EMT in hepatocellular carcinoma [28]. To determine the effect of HGF on EMT and metastasis of lung cancer, we examined the morphology, EMT markers expression, migration and invasive abilities of lung cancer cells after HGF stimulation. Lung adenocarcinoma A549 cells and large cell lung cancer 95D cells which express higher levels of HGF receptor protein (c-Met) are used in the present study. The results were shown in Figure 2A, both of two types of cells treated with HGF (50 ng/ml) for 72 h underwent typical EMT morphological changes, acquired a spindle-shaped and fbroblast-like phenotype. In contrast, A549 and 95D cells pretreated with c-Met inhibitors, SU11274, inhibited HGF-induced morphologic changes and showed a larger, flattened phenotype (Figure 2A). While western blotting demonstrated that the expression of E-cadherin protein (epithelial marker) was significantly downregulated, and vimentin protein (mesenchymal marker) was substantially upregulated in the HGF-treated cells compared to the control cells (Figure 2B). However, SU11274 reversed the HGF-induced downregulation of E-cadherin and upregulation of vimentin (Figure 2B). In addition, wound-healing and transwell assays showed that HGF-stimulated A549 and 95D cells also acquired an increase of the migratory and invasive capacity, and the effect can also be inhibited by SU11274 (Figure 2C, 2D). Furthermore, it's worth mentioning that pretreatment with HGF-blocking antibodies, a commercial antibodies certified for blocking the HGF-receptor interaction, presented the similar results with SU11274 (data not shown).

### miR-206 attenuates HGF-induced EMT, migration and invasion of lung cancer cells

**Figure 3:**
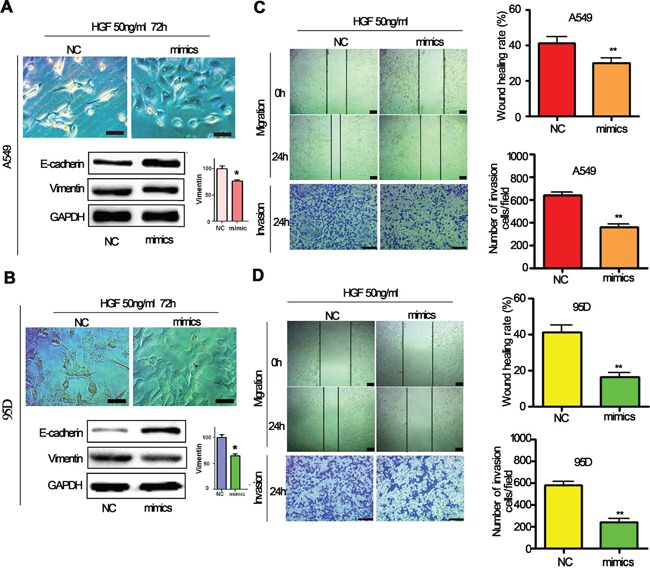
HGF-induced EMT was inhibited by miR-206 mimics **A, B.** A549 cells and 95D cells were transfected with miR-206 mimics and then stimulated with 50 ng/mL of HGF, epithelial morphology were observed under a light microscope (Bar = 50μm). Meanwhile, western blot analysis showed that miR-206 mimics treatment resulted in high expression of E-cadherin and low expression of vimentin **C, D.** HGF induced migration and invasion were inhibited by miR-206 mimics. A549 cells and 95D cells were transfected with miR-206 mimics or control sequences, and then treated with 50 ng/mL of HGF. Wound-healing assays and transwell invasion assays were used to investigate the migratory and invasive ability of cells, respectively. Cells were transfected with miR-206 mimics treatment led to inhibition of migration and invasion induced by HGF exposure. Bar = 200μm.* *P*<0.05, ** *P*<0.01 vs control.

Since it has been demonstrated that HGF induces EMT, migration and invasion of lung cancer cells, we next determined whether miR-206 regulates HGF-induced EMT, migration and invasion of lung cancer cells. A549 cells and 95D cells were transfected with miR-206 mimics and negative control (NC) and then exposed to 50 ng/mL of HGF for 72 hours. We found that transfection of miR-206 mimics inhibited the conversion from epithelial phenotype to mesenchymal phenotype after HGF stimulation (Figure 3A, 3B). Western blotting analysis showed that A549 and 95D cells transfected with miR-206 mimics after HGF stimulation had increased expression of E-cadherin and decreased expression of vimentin (Figure 3A, 3B). EMT is associated with enhanced cell motility. To examine whether miR-206 affects the invasion of lungcancer cells stimulated with HGF, wound-healing and transwells assays were further performed. We observed that miR-206 mimics also significantly inhibited HGF induced cell migration and invasion (Figure 3C, 3D). These results suggested that miR-206 could block HGF induced EMT, migration and invasion of human lung cancer cells.

### MiR-206 regulates HGF-induced lung cancer cell EMT, migration and invasion through c-Met/PI3k/Akt/mTOR signaling

**Figure 4:**
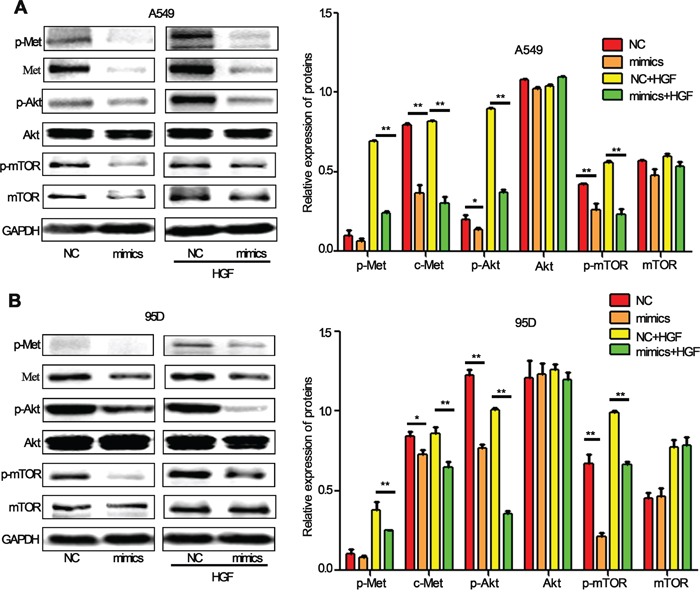
miR-206 inhibited HGF-induced activation of PI3k/Akt/mTOR signaling in lung cancer cells **A, B.** Western blot analysis showed that miR-206 downregulated the expression of c-Met, p-Met, p-Akt (ser473) and p-mTOR proteins. Under HGF (20 ng/ml, 15min) stimulation, miR-206 showed more obviously inhibitory effect on PI3k/Akt/mTOR pathway both in A549 cells and 95D cells. Relative protein expression was calculated by gray value of indicated protein/GAPDH. NC: Negative control. * *P*<0.05, ** *P*<0.01 vs its control as indicated in Figure.

Identification of miRNA-regulated gene targets is a necessary step to understand miRNA functions. It has been reported that HGF/c-Met signaling is important in EMT and metastasis and its downstream PI3k/Akt/mTOR signaling is one of the major pathways activated in cancer cells, including lung cancer cells [29, 30]. In our previous study, we have demonstrated that c-Met could be a direct target of miR-206 in cancer cells [31]. We therefore sought to validate whether miR-206 inhibited HGF-induced EMT, migration and invasion by c-Met PI3k/Akt/mTOR pathway. As expected, miR-206 mimics transfection significantly decrease the expression of c-Met, p-Akt and p-mTOR in both A549 and 95D cells (Figure 4). Furthermore, inhibitory effects of miR-206 on c-Met/Akt/mTOR pathway were more obvious in HGF-stimulated A549 cells and 95D cells.

**Figure 5:**
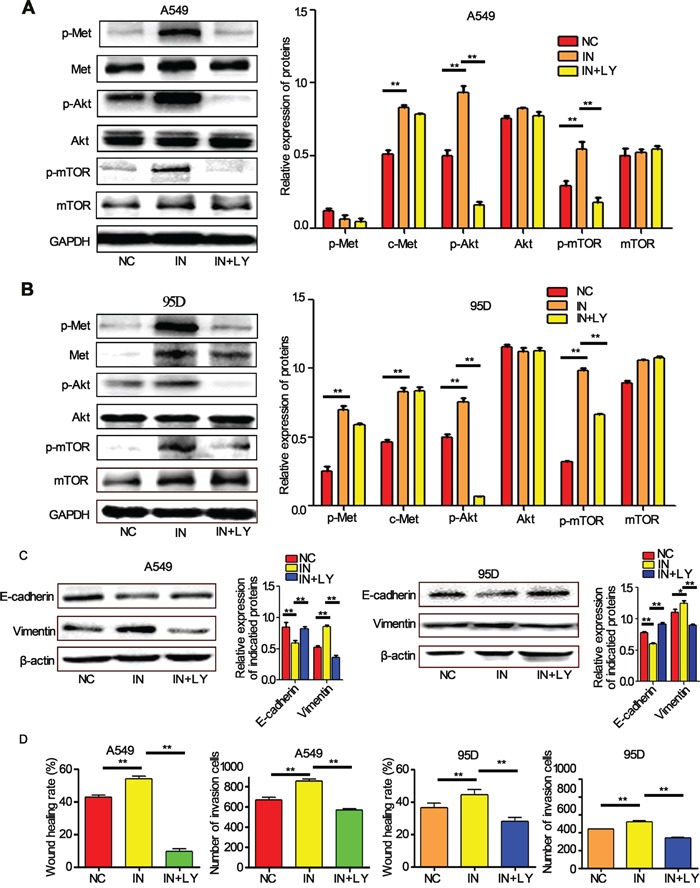
miR-206 inhibitors increased the EMT, migration and invasion of cells **A, B.** The expression levels of p-c-Met, c-Met, p-Akt, Akt, p-mTOR, mTOR in A549 cells and 95D cells detected by western blots. miR-206 inhibitors significantly increased the expression of these proteins (except mTOR in A549 cells) compared with the negative control (NC). PI3k inhibitor LY294002 decreased the expression levels of all these proteins except Met and mTOR. **C, D.** LY294002 inhibited miR-206 inhibitor-induced EMT, migration and invasion both in A549 and 95D cells. ** P<0.01 vs its control as indicated in Figure. NC: Negative control; IN: miR-206 inhibitors; LY: LY294002.

**Figure 6:**
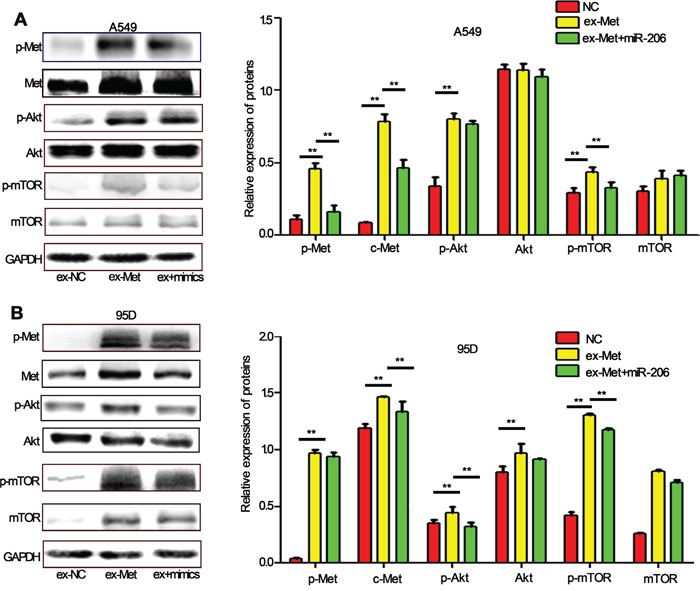
miR-206 inhibits c-Met overexpression-induced activation of Akt/mTOR pathway **A, B.** A549 cells (A) and 95D cells (B) were transfected with c-Met overexpression plasmid (ex-Met) or control (ex-NC). 48 h after transfection, the cells were collected. The expression of p-c-Met, c-Met, p-Akt, Akt, p-mTOR, mTOR was analyzed by western blot assay. The results showed that the expression levels of these proteins were increased significantly (except Akt in A549 cells). Cotransfected with miR-206 mimics and c-Met overexpression plasmid significantly decreased the expression of p-Met, Met, p-Akt, Akt, p-mTOR. Relative protein expression was calculated by gray value of indicated protein/GAPDH. ** *P*<0.01 vs its control as indicated in Figure.

Further investigation showed that the knockdown of miR-206 expression by its inhibitors induced a sharp activation of c-Met/Akt/mTOR pathway. However, the effects were suppressed by LY294002, a PI3k specific inhibitor, which can significantly reduce phosphorylation of Akt and mTOR (Figure 5A, 5B). Furthermore, we found miR-206 inhibitors induced EMT, migration and invasion both in A549 and 95D cells, and the effects could also be attenuated by LY294002 (Figure 5C, 5D). To further evaluate the relationship between miR-206 and the c-Met/Akt/mTOR pathway, we transiently transfected A549 and 95D cells with c-Met overexpression plasmid (ex-Met). We found that ex-Met plasmid dramatically increased the expression of p-Met, c-Met, p-Akt and p-mTOR compared with the negative control (ex-NC) group (Figure 6A). While cotransfection with miR-206 mimics resulted in a significant reduction in total c-Met expression and phosphorylation levels of c-Met/Akt/mTOR pathway proteins (Figure 6A, 6B). Taken together, miR-206 inhibited HGF-induced EMT through mediated HGF/c-Met dependent Akt/mTOR signaling pathways.

### MiR-206 inhibits HGF-induced migration and capillary tube formation of HUVEC cells through c-Met/PI3k/Akt/mTOR pathway

**Figure 7:**
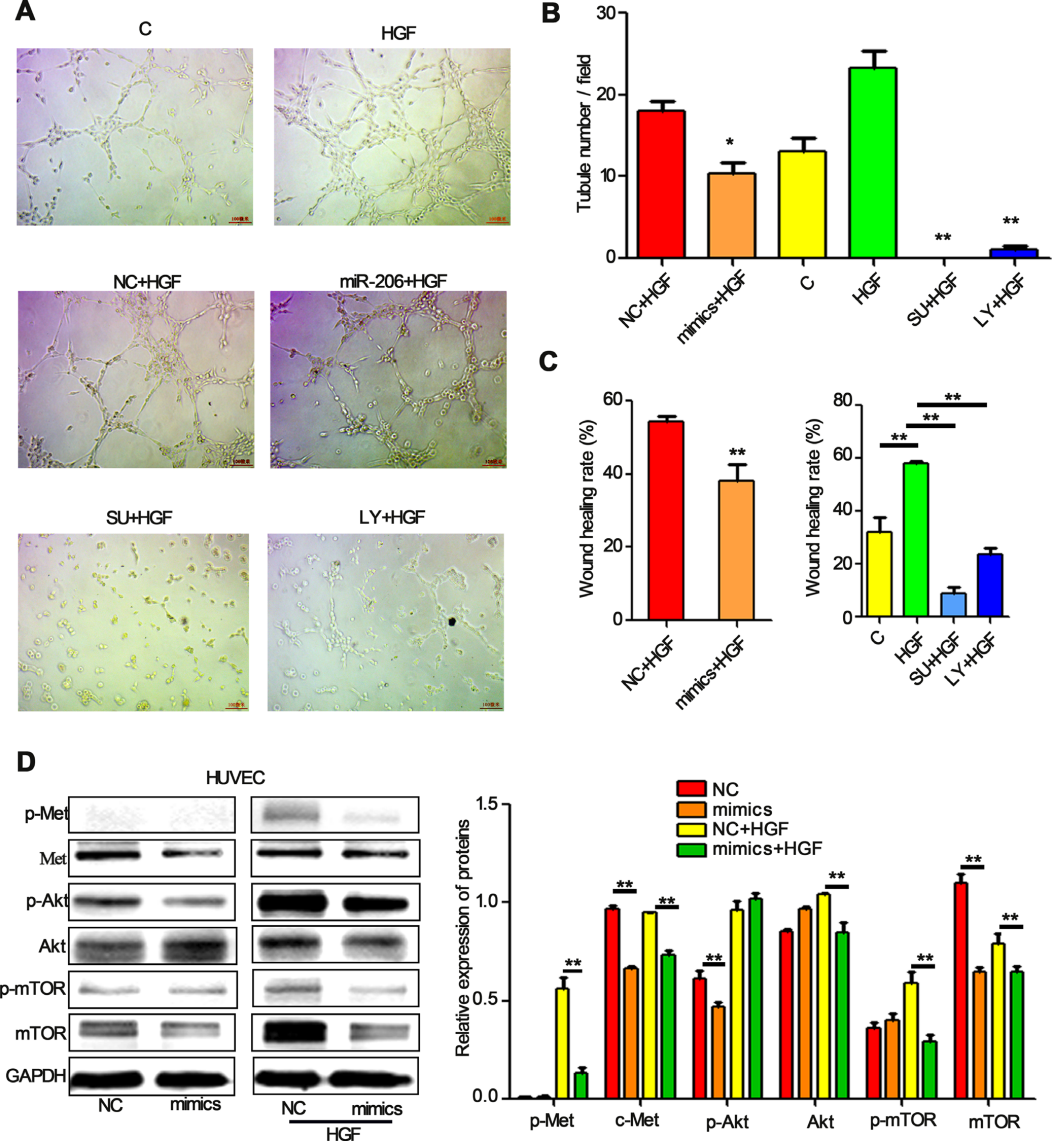
miR-206 suppresses the migration and tube formation of HUVEC cells **A, B.** Representation (A) and quantification (B) of tube formation assay showing the angiogenic capability of HUVECs transfected with NC or miR-206, and then stimulated with HGF (50 ng/ml). Original magnification, ×100. * *P*<0.05 vs NC+HGF, ** *P*<0.01 vs HGF **C.** Quantification of scratch migration assay showing the migration of HUVEC cells transfected with NC or miR-206, and then stimulated with HGF (50 ng/ml). **D.** HUVECs transfected with NC or miR-206 were stimulated with 20 ng/ml of HGF for 15min. The cells were then harvested and lysed for the detection of p-c-Met, c-Met, p-Akt, Akt, p-mTOR, mTOR, and GAPDH. C: control; NC: Negative control; SU: SU11274; LY: LY294002 ** *P*<0.01.

Angiogenesis is a critical step in solid tumor progression, and is also a crucial aspect of endothelial cell biology. To understand the function of miR-206 in angiogenesis, we further determined the effects of miR-206 on the abilities of in vitro capillary tube formation of HUVECs. HUVECs were transfected with miR-206 mimics and seeded on matrigel at low density, and the formation of tubules in the presence of HGF was characterized. HGF significantly induced in vitro capillary tube formation of HUVECs, and which could be completely abolished by c-Met inhibitor (SU11274) and LY294002 (Figure 7A, 7B). Meanwhile, miR-206 mimics transfection led to a 42.59 % of the decrease in the number of tubes in miR-206 mimics plus HGF group compared to its negative control group (Figure 7A, 7B). In addition, wound healing assay results also showed that miR-206 mimics significantly inhibited wound closure of HUVECs (Figure 7C). These data suggest that upregulation of miR-206 may block migration and capillary tube formation of HUVEC cells and therefore inhibited metastasis of lung cancers.

The PI3k/Akt/mTOR and Hypoxia-inducible factor-1alpha (HIF-1α)-VEGF signaling pathway have been found to be involved in HGF-inducing VEGF expression [32]. Accordingly, we next investigated whether miR-206 suppressed tube formation of HUVECs by PI3k/Akt/mTOR pathway. Indeed, transfection with miR-206 attenuated the expression of p-Met, Met, p-Akt and p-mTOR both in HUVEC cells and HGF stimulated HUVEC cells (Figure 7D). These results indicate that miR-206 suppresses capillary tube formation of HUVEC cells by inhibiting the HGF-c-Met signaling, and then influencing the downstream Akt/mTOR signaling pathways.

### MiR-206 inhibited lung cancer growth, metastasis and angiogenesis in tumor xenografts

**Figure 8:**
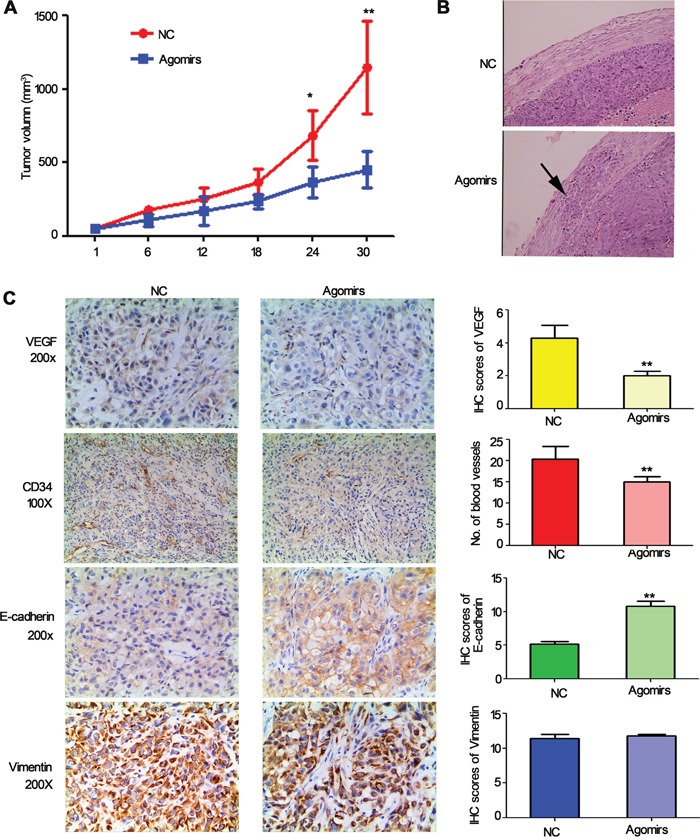
The effect of miR-206 on tumor growth, metastasis and angiogenesis and EMT in tumour xenografts **A.** Tumor volumes of nude mice treated with miR-206 agomirs or NC. NC: Negative control. *P < 0.05, **P < 0.01 vs NC. **B.** H&E staining showed that the tumors and tissues in miR-206 agomirs injected groups had clear boundaries with less invasiveness. Original magnification, ×100. **C.** Expression of VEGF, CD34, E-cadherin, vimentin in tumor tissues by immunohistochemistry.

To further determine whether miR-206 is involved in tumorigenesis *in vivo*, a xenograft tumor model was used in the nude mice. Because miR-206 is down-regulated in lung cancer cells, miR-206 agomirs (more stable than miR-206 mimics) were used to generate a gain-of-function model. As a result, the volume of tumors derived from the miR-206 agomirs-treated groups was dramatically reduced compared to the control group with 30 days of treatment (Figure 8A). H&E staining showed that the tumors tissues in miR-206 agomirs groups had clear boundaries with less invasiveness. In contrast, tumor tissues arising from control groups displayed characteristics of invasion (Figure 8B), indicating that miR-206 inhibited invasive and metastasis of lung cancer *in vivo*.

To analyze angiogenesis and EMT of tumors, tumor tissues were analysed by immunohistochemical staining with CD34, VEGF, E-cadherin and vimentin antibodies. The results indicated that the expression of VEGF and MVD in the miR-206 agomirs group was slighter compared to the control vector group (Figure 8C). Furthermore, the expression of E-cadherin in the miR-206 agomirs group was significantly higher than that of the control vector group, while the expression of vimentin in the miR-206 agomirs group was slightly lower than that of the control vector group. Taken together, these data indicated that the expression of miR-206 greatly inhibited the process of tumor progression *in vivo*, and miR-206 would seem to regulate tumourigenesis and metastasis via inhibiting EMT and VEGF-mediated angiogenesis.

## DISCUSSION

EMT and angiogenesis are associated with lung cancer development and metastasis [3]. Although recent evidences showed that altered miR-206 expression was implicated in the occurrence of metastasis of lung cancer. However, whether miR-206 contributes EMT, angiogenesis and the molecular mechanisms remain elusive. In this study, we revealed that enhanced expression of miR-206 could reverse HGF-induced EMT and angiogenesis in lung cancer through inhibiting c-Met/Akt/mTOR pathway. Our findings further highlighted the role of miR-206 downregulation in promoting lung cancer development and metastasis, implicating that miR-206 could be a potential candidate for lung cancer therapy.

HGF is a multifunctional cytokine that binds to the c-Met proto-oncogene to promote cell proliferation, cell survival, cell migration, branching morphogenesis and invasion in a variety of systems [33]. Accumulated evidence suggested that HGF plays the role of an EMT regulator and accelerates the tumor-promoting activity in various cancer progressions involving the progression of metastatic lung cancer [34–37]. Consistent with previous reports, we found that HGF stimulated the phosphorylation of c-Met followed by an increase in the levels of vimentin and decrease of E-cadherin in lung cancer A549 and 95D cells. Furthermore, HGF-treated A549 and 95D cells have undergone EMT and exhibited enhanced invasiveness and motility, and c-Met inhibitors, SU11274 can suppress the effects of HGF, suggesting HGF/c-Met signaling pathway could be a therapeutic target of lung cancers.

Increasing evidences have confirmed that miR-206 acted as a tumor-suppressor in various cancers including lung cancer [17-22, 38]. Although multiple anti-cancer functions of miR-206 have been reported, its underlying anti-tumor mechanisms of action are not yet fully understood. Recent studies have implied that miRNAs act as crucial modulators for EMT [39–41]. For example, Downregulation of miR-638 promotes invasion and proliferation by regulating SOX2 and induces EMT in NSCLC [39]. In addition, miR-134 inhibits epithelial to mesenchymal transition by targeting FOXM1 (forkhead box protein M1) in non-small cell lung cancer cells [40]. Moreover, microRNA-451 induces EMT in docetaxel-resistant lung adenocarcinoma cells by targeting proto-oncogene c-Myc [41]. In this study, we found that ectopic expression of miR-206 inhibited HGF-induced EMT, leading to suppression of HGF-induced invasion and migration in lung cancer cells. In contrast, miR-206 inhibitors reversed this process, suggesting that miR-206 could be a novel repressor of the EMT in lung cancer cells.

It is known that HGF/c-Met signals via a number of intracellular signaling mechanisms, including PI3k/Akt, Ras/MAPK (mitogen activated protein kinase) and the JAK/STAT (Janus tyrosine kinase/Signal transducer and activator of transcription) pathway, to increase scattering/motility, invasion, proliferation, survival and morphogenesis [42, 43]. The interaction of PI3k with activated c-Met may enhance PI3k activity, which has been implicated in the form of EMT and cell motility [30]. It has been reported that miR-206 activates apoptosis and inhibits tumor cell proliferation, migration and colony formation through targeting c-Met and Bcl-2 [44]. Consistently, in this study, we suggested that miR-206 suppressed HGF-induced EMT, cell migration, and invasion via by targeting c-Met and its downstream Akt/mTOR signaling pathways.

Tumor angiogenesis is important for tumor progression, including metastasis. The migration, invasion and tube formation of endothelial cells (ECs) are important for tumor angiogenesis. Some reports have shown that miR-26a, miR-132 and miR-107 regulate endothelial cell functions and affect blood vessel formation and extension [32, 45, 46]. In this study, we found the important roles of miR-206 in inhibiting angiogenesis, which is supported by a number of *in vitro* and *in vivo* experiments. We also observed that miR-206 inhibited HUVEC migration, and tube formation *in vitro* and increased micro-vessel density *in vivo* partly through c-Met/PI3k/Akt/mTOR signaling pathways. These results suggested that the inhibitory effects of miR-206 on angiogenesis are related to PI3k/Akt/mTOR signaling pathways.

**Figure 9:**
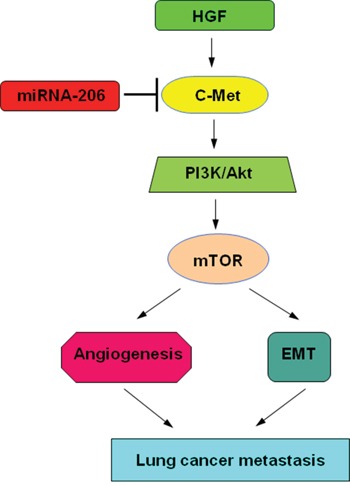
Proposed models on the inhibitory role of miR-206 in HGF-induced EMT, angiogenesis and metastasis As depicted in the model, miR-206 targets c-Met and through PI3k/Akt/mTOR signaling cascade modulates: 1) Angiogenesis of HUVECs. 2) Epithelial-mesenchymal transition (EMT) of lung cancer cells, finally affecting lung cancer metastasis.

In conclusion, overexpression of miR-206 could not only inhibit HGF-induced EMT, migration and invasion of lung cancer cells, but also reduce migration and tube formation of HUVECs. Targeting c-Met by miR-206 and subsequent inhibiting PI3k/Akt/mTOR signaling axis play an important roles in these processes (Figure 9). Also in mice xenograft tumor model, miR-206 showed a significant inhibitory effect on lung cancer growth, EMT and angiogenesis. Therefore, miR-206 might be a potential target for the therapeutic strategy against EMT and angiogenesis of lung cancer.

## MATERIALS AND METHODS

### Cell lines and tissue samples

Human lung cancer cell lines (A549, 95D, 95C, 801D), human normal bronchial epithelial cell line (BEAS-2B) and human umbilical vein endothelial cells (HUVECs), were purchased from Chinese Academy of Sciences Cell Bank (Shanghai, China). All the cell lines were maintained in a 37°C, 5% CO_2_ incubator in RPMI-1640 medium supplemented with 10% fetal bovine serum (FBS). 35 fresh frozen tumor tissue samples (35 adenocarcinoma) and corresponding non-tumor lung tissue samples were obtained after informed consent from the patients in the Department of Respiratory Disease of the 117th Hospital of PLA. None of these patients received chemotherapy and radiotherapy before the surgery.

### Growth factors, inhibitors and antibodies

HGF was purchased from Peprotech (Shanghai, China). c-Met inhibitor (SU11274) and PI3 kinase inhibitor (LY294002) were purchased from Selleck Chemicals (Shanghai, China). HGF antibody was purchased from R&D Systems (Shanghai, China). Anti-mTOR antibody (2972), anti-pSer2481-mTOR antibody (2974), anti-phospho-Ser473-Akt antibody (4051), anti-Akt totol antibody (4691), anti-E-cadherin antibody (3195), anti-vimentin (5741), anti-Met antibody (3148), anti-phospho-Met antibody (3077), anti-GAPDH antibody (5174), and anti-β-actin antibody (3700) were purchased from Cell Signaling Technology (Danvers, MA), All these antibodies used 1: 1000 dilution in this study.

### MiRNA and c-Met overexpression vector

MiR-206 mimics, inhibitors and corresponding controls were chemically synthesized by GenePharma Inc (Shanghai, China). Sequences of miR-206 mimics, inhibitor and corresponding controls were showed as follows: miR-206 mimics: S: 5′-UGG AAG UAA GGA AGU GUG UGG-3′; A:5′-ACA CAC UUC CUU ACA UUC CAU U-3′;Mimics negative control: S:5′-UUC UCC GAA CGU GUC ACG UTT-3′; A:5′-ACG UGA CAC GUU CGG AGA ATT-3′; miR-206 inhibitors: 5′-CCA CAC ACU UCC UUA CAU UCC A – 3′; Inhibitors negative control: 5′-CAG UAC UUU UGU GUA GUA CAA-3′. c-Met (Accession NO.: NM_000245) overexpression vector (pEZ/M98/neo-c-Met, designated as ex-Met) and the control vector (pEZ/M98/neo-control, designated as ex-NC) were provided by genecopoeia Inc (Guangzhou, China).

### Quantitative real-time PCR

Quantitative real-time PCR (qRT-PCR) was used to determine the miR-206 expression. The primers for RT-PCR were designed based on the miR-206 sequences provided by the Sanger Center miRNA Registry. The RT primers were designed as follows: RT primer: CTC AGC GGC TGT CGT GGA CTG CGC GCT GCC GCT GAG CCA CAC AC. PCR primers: F: GGC GGT GGA ATG TAA GGA AG; R: GGC TGT CGT GGA CTG CG. The qRT-PCR was performed on the ABI (Applied Biosystems) 7900 HT Thermal cycler in standard mode for 40 cycles. Relative miRNA expression values (Target miRNA vs U6) were calculated with the 2 ^−ΔΔCt^ method.

### Cell transfection

Both miRNA-206 mimics or inhibitors and overexpression vectors were transfected by using Lipofectamine 2000 (Invitrogen, Shanghai, China) reagent according to the manufacturer's instructions as described previously [47]. At 6 h after transfection, the medium was replaced. The total proteins were extracted 48 h later for the further tests.

### Luciferase reporter assay

Luciferase reporter constructs containing portions (position: 2030) of the c-Met 3′-UTR (pmirGLO-c-Met-wt), mutant sequence (pmirGLO–c-Met–mut) and miR-206 inhibitor sequence (Positive control, pmir GLO–c-Met–PC) were generated by GenePharma Inc (Shanghai, China). A549 cells were cultured in 24-well plates for 24 hours and cotransfected with 25 ng of c-Met 3′-UTR reporter constructs and 20 nM of miR-206 mimicsor mimic-NC using lipofectamine 2000 for 24 hours. After transfection, cells were harvested, lysed, and assayed with the Dual-Luciferase Reporter Assay Kit (Promega) according to the manufacturer's instructions. Firefly luciferase activity was normalized to renilla luciferase activity for each transfected well. Each experiment was performed in duplicate and repeated three times.

### Wound healing assay

Wound healing assay was conducted to examine the capacity of cell migration. Briefly, the wound was generated when the cells reached 90-95% confluent in a 12-well plate by scratching the surface of the plates with 10 μl pipette tip. The cells were then incubated in 2.5% FBS for 24h, and then photographed using phase-contrast microscopy (Leica). The distance between the wound edges of the scratch area was analyzed using Adobe Photoshop 7.0. All experiments were performed in triplicate.

### Cell invasion assay

The invasive potential of cells was measured in transwell insert with 8.0 μm pore polycarbonate membrane (Corning) according to the manufacturer's instructions. The filter of top chamber was matrigel-coated with 50 μl of diluted matrigel following the standard procedure and incubated at 37°C for 2 h. The lower chambers were filled with 600 μl of RPMI 1640 medium containing 10 % FBS as chemoattractant. Then 50,000 cells in 100μl serum-free medium were added into each top chamber. After the cells were incubated for 24h, the non-invading cells that remained on the upper surface were removed with a cotton swab. The invasive cells on the lower surface of the membrane insert were fixed with cold methanol for 45 min, and then stained with 0.1% crystal violet for 30 min. The number of cells on the lower surface, which had invaded through the membrane, was counted under a light microscope in five random fields at a magnification of 100×. The experiments were repeated three times independently.

### Capillary tube formation analyses

HUVECs were transfected with a miR-206 mimics or negative control, then the tranfected cells (5 ×10^3^/well) were further cultured at 37°C for 12 hours in a 96-well plate coated with Matrigel (BD Pharmingen). The formation of capillary-like structures was captured under a light microscope. The number of the formed tubes, which represent the degree of angiogenesis in vitro, was scanned and quantitated in five low power fields (100× magnification).

### Western blot analysis

The whole-cell extracts were prepared in RIPA buffer (20 mM Tris, 2.5 mM EDTA, 1% Triton X-100, 1% deoxycholate, 0.1% SDS, 40 mM NaF, 10 mM Na4P_2_O_7_ and 1 mM PMSF). Thirty micrograms cellular protein of each sample was applied to immunoblot following 10% SDS-PAGE electrophoresis and probed with indicated antibodies (MET antibody, Abcam company), followed by a horseradish peroxidase-conjugated goat anti-mouse or anti-rabbit antibody (Millipore). Immunoreactive bands were visualized by enhanced chemiluminescence (Millipore) according to the manufacturer's instructions. Quantification of reactive protein bands was performed by densitometric analysis and the fold change was calculated by normalizing with β-actin or GAPDH levels.

### Animal studies

All experimental procedures involving animals in this study had been approved by the ethics committee in the 117th Hospital of PLA. Male nude mice (BALB/c, 4 weeks old) were purchased from Shanghai Laboratory Animal Center (Shanghai, China). For preparation of subcutaneous xenograft model, 0.2 ml A549 lung cancer cells (2.0×10^6^) were injected subcutaneously into the right flank of the nude mice. 15 days after tumor cell inoculation with confirmation of successful maturation of tumors, 20 mice were divided randomly into four groups (five mice per group). miR-206 agomirs and miR-206 agomir negative control (NC) (2 nmol; Genepharma, Shanghai, China) were given locally by direct injection into the xenografts every three days. Meanwhile, tumor volumes were determined (in cubic millimeter) by measuring in two directions and was calculated as tumor volume = length × (width)^2^/2. After 30 days of treatment, all mice were sacrificed. Transplanted tumors were excised and divided into two parts. One part was stored in −80°C for further use. And another section was fixed in formalin, and embedded in paraffin. Vascular endothelial growth factor (VEGF) and CD34 were detected in tumor tissues by immunohistochemistry. The microvessel density (MVD) in tumor tissues was evaluated based on the staining for CD34. 10 random fields per tumor sample at 200 × were captured and quantified as CD34-positive area/total area by Image-Pro Plus software (Media Cybernetics, Inc.).

### Immunohistochemistry

Tissue slides were incubated for 2 h at 56°C and de-paraffinized. Antigen retrieval was obtained by microwave treatment in citrate buffer for 15 min to retrieve antigenicity. After peroxidase activity was blocked with 3% H_2_O_2_/methanol for 10 min, sections were incubated with normal goat serum for 20 min to block non-specific antibody binding sites. Sections were incubated with the primary antibodies for 1 h at 25°C followed by incubations with biotinylated anti-rabbit/mouse IgG and peroxidase-labelled streptavidin for 10 min each. No specific staining was observed in the negative control slides prepared without primary antibody.

### Statistical analysis

All statistical analyses were performed using SPSS 13.0. Data were expressed as mean ± SD. The statistical difference of data between groups was analyzed by one-way analysis of variance (ANOVA) and Student's t test. Statistical significance was concluded at *P* < 0.05.

## References

[1] Torre LA, Bray F, Siegel RL, Ferlay J, Lortet-Tieulent J, Jemal A (2015). Global cancer statistics, 2012. CA: a cancer journal for clinicians.

[2] Chaffer CL, Weinberg RA (2011). A perspective on cancer cell metastasis. Science.

[3] Iwatsuki M, Mimori K, Yokobori T, Ishi H, Beppu T, Nakamori S, Baba H, Mori M (2010). Epithelial-mesenchymal transition in cancer development and its clinical significance. Cancer science.

[4] Meng F, Wu G (2012). The rejuvenated scenario of epithelial-mesenchymal transition (EMT) and cancer metastasis. Cancer metastasis reviews.

[5] Fakhrejahani E, Toi M (2012). Tumor angiogenesis: pericytes and maturation are not to be ignored. Journal of oncology.

[6] Griffioen AW (2007). Therapeutic approaches of angiogenesis inhibition: are we tackling the problem at the right level?. Trends in cardiovascular medicine.

[7] Kong W, He L, Richards EJ, Challa S, Xu CX, Permuth-Wey J, Lancaster JM, Coppola D, Sellers TA, Djeu JY, Cheng JQ (2014). Upregulation of miRNA-155 promotes tumour angiogenesis by targeting VHL and is associated with poor prognosis and triple-negative breast cancer. Oncogene.

[8] Gao D, Vahdat LT, Wong S, Chang JC, Mittal V (2012). Microenvironmental regulation of epithelial-mesenchymal transitions in cancer. Cancer research.

[9] Gentile A, Trusolino L, Comoglio PM (2008). The Met tyrosine kinase receptor in development and cancer. Cancer metastasis reviews.

[10] Maemura M, Iino Y, Yokoe T, Horiguchi J, Takei H, Koibuchi Y, Horii Y, Takeyoshi I, Ohwada S, Morishita Y (1998). Serum concentration of hepatocyte growth factor in patients with metastatic breast cancer. Cancer letters.

[11] Feng Y, Minca EC, Lanigan C, Liu A, Zhang W, Yin L, Pennell NA, Farver C, Tubbs R, Ma PC (2014). High MET receptor expression but not gene amplification in ALK 2p23 rearrangement positive non-small-cell lung cancer. Journal of thoracic oncology.

[12] Umeguchi H, Sueoka-Aragane N, Kobayashi N, Nakamura T, Sato A, Takeda Y, Hayashi S, Sueoka E, Kimura S (2015). Usefulness of plasma HGF level for monitoring acquired resistance to EGFR tyrosine kinase inhibitors in non-small cell lung cancer. Oncology reports.

[13] Huang J, Hao P, Chen H, Hu W, Yan Q, Liu F, Han ZG (2009). Genome-wide identification of Schistosoma japonicum microRNAs using a deep-sequencing approach. PloS one.

[14] Yao Q, Xu H, Zhang QQ, Zhou H, Qu LH (2009). MicroRNA-21 promotes cell proliferation and down-regulates the expression of programmed cell death 4 (PDCD4) in HeLa cervical carcinoma cells. Biochemical and biophysical research communications.

[15] Ohta K, Hoshino H, Wang J, Ono S, Iida Y, Hata K, Huang SK, Colquhoun S, Hoon DS (2015). MicroRNA-93 activates c-Met/PI3K/Akt pathway activity in hepatocellular carcinoma by directly inhibiting PTEN and CDKN1A. Oncotarget.

[16] Bai J, Mei P, Zhang C, Chen F, Li C, Pan Z, Liu H, Zheng J (2013). BRG1 is a prognostic marker and potential therapeutic target in human breast cancer. PloS one.

[17] Yang Q, Zhang C, Huang B, Li H, Zhang R, Huang Y, Wang J (2013). Downregulation of microRNA-206 is a potent prognostic marker for patients with gastric cancer. European journal of gastroenterology & hepatology.

[18] Ren J, Huang HJ, Gong Y, Yue S, Tang LM, Cheng SY (2014). MicroRNA-206 suppresses gastric cancer cell growth and metastasis. Cell & bioscience.

[19] Wang S, Lu S, Geng S, Ma S, Liang Z, Jiao B (2014). Decreased expression of microRNA-206 correlates with poor clinical outcome in patients with malignant astrocytomas. Pathology oncology research : POR.

[20] Chen X, Yan Q, Li S, Zhou L, Yang H, Yang Y, Liu X, Wan X (2012). Expression of the tumor suppressor miR-206 is associated with cellular proliferative inhibition and impairs invasion in ERalpha-positive endometrioid adenocarcinoma. Cancer letters.

[21] Yan D, Dong Xda E, Chen X, Wang L, Lu C, Wang J, Qu J, Tu L (2009). MicroRNA-1/206 targets c-Met and inhibits rhabdomyosarcoma development. The Journal of biological chemistry.

[22] Zhang Y, Tang H, Cai J, Zhang T, Guo J, Feng D, Wang Z (2011). Ovarian cancer-associated fibroblasts contribute to epithelial ovarian carcinoma metastasis by promoting angiogenesis, lymphangiogenesis and tumor cell invasion. Cancer letters.

[23] Nohata N, Hanazawa T, Enokida H, Seki N (2012). microRNA-1/133a and microRNA-206/133b clusters: dysregulation and functional roles in human cancers. Oncotarget.

[24] Liu H, Cao YD, Ye WX, Sun YY (2010). Effect of microRNA-206 on cytoskeleton remodelling by downregulating Cdc42 in MDA-MB-231 cells. Tumori.

[25] Zhang T, Liu M, Wang C, Lin C, Sun Y, Jin D (2011). Down-regulation of MiR-206 promotes proliferation and invasion of laryngeal cancer by regulating VEGF expression. Anticancer research.

[26] Peng Y, Guo JJ, Liu YM, Wu XL (2014). MicroRNA-34A inhibits the growth, invasion and metastasis of gastric cancer by targeting PDGFR and MET expression. Bioscience reports.

[27] Elliman SJ, Howley BV, Mehta DS, Fearnhead HO, Kemp DM, Barkley LR (2014). Selective repression of the oncogene cyclin D1 by the tumor suppressor miR-206 in cancers. Oncogenesis.

[28] Nagai T, Arao T, Furuta K, Sakai K, Kudo K, Kaneda H, Tamura D, Aomatsu K, Kimura H, Fujita Y, Matsumoto K, Saijo N, Kudo M, Nishio K (2011). Sorafenib inhibits the hepatocyte growth factor-mediated epithelial mesenchymal transition in hepatocellular carcinoma. Molecular cancer therapeutics.

[29] Birchmeier C, Birchmeier W, Gherardi E, Vande Woude GF (2003). Met, metastasis, motility and more. Nature reviews Molecular cell biology.

[30] Ma PC, Jagadeeswaran R, Jagadeesh S, Tretiakova MS, Nallasura V, Fox EA, Hansen M, Schaefer E, Naoki K, Lader A, Richards W, Sugarbaker D, Husain AN, Christensen JG, Salgia R (2005). Functional expression and mutations of c-Met and its therapeutic inhibition with SU11274 and small interfering RNA in non-small cell lung cancer. Cancer research.

[31] Chen QY, Jiao DM, Yan L, Wu YQ, Hu HZ, Song J, Yan J, Wu LJ, Xu LQ, Shi JG (2015). Comprehensive gene and microRNA expression profiling reveals miR-206 inhibits MET in lung cancer metastasis. Molecular bioSystems.

[32] Yang X, Zhang XF, Lu X, Jia HL, Liang L, Dong QZ, Ye QH, Qin LX (2014). MicroRNA-26a suppresses angiogenesis in human hepatocellular carcinoma by targeting hepatocyte growth factor-cMet pathway. Hepatology.

[33] Whittaker S, Marais R, Zhu AX (2010). The role of signaling pathways in the development and treatment of hepatocellular carcinoma. Oncogene.

[34] Matsumoto K, Nakamura T (1996). Emerging multipotent aspects of hepatocyte growth factor. Journal of biochemistry.

[35] Siegfried JM, Weissfeld LA, Luketich JD, Weyant RJ, Gubish CT, Landreneau RJ (1998). The clinical significance of hepatocyte growth factor for non-small cell lung cancer. The Annals of thoracic surgery.

[36] Bharti A, Ma PC, Maulik G, Singh R, Khan E, Skarin AT, Salgia R (2004). Haptoglobin alpha-subunit and hepatocyte growth factor can potentially serve as serum tumor biomarkers in small cell lung cancer. Anticancer research.

[37] Olivero M, Rizzo M, Madeddu R, Casadio C, Pennacchietti S, Nicotra MR, Prat M, Maggi G, Arena N, Natali PG, Comoglio PM, Di Renzo MF (1996). Overexpression and activation of hepatocyte growth factor/scatter factor in human non-small-cell lung carcinomas. British journal of cancer.

[38] Zheng Z, Yan D, Chen X, Huang H, Chen K, Li G, Zhou L, Zheng D, Tu L, Dong XD (2015). MicroRNA-206: Effective Inhibition of Gastric Cancer Progression through the c-Met Pathway. PloS one.

[39] Xia Y, Wu Y, Liu B, Wang P, Chen Y (2014). Downregulation of miR-638 promotes invasion and proliferation by regulating SOX2 and induces EMT in NSCLC. FEBS letters.

[40] Li J, Wang Y, Luo J, Fu Z, Ying J, Yu Y, Yu W (2012). miR-134 inhibits epithelial to mesenchymal transition by targeting FOXM1 in non-small cell lung cancer cells. FEBS letters.

[41] Chen D, Huang J, Zhang K, Pan B, Chen J, De W, Wang R, Chen L (2014). MicroRNA-451 induces epithelial-mesenchymal transition in docetaxel-resistant lung adenocarcinoma cells by targeting proto-oncogene c-Myc. European journal of cancer.

[42] Trusolino L, Comoglio PM (2002). Scatter-factor and semaphorin receptors: cell signalling for invasive growth. Nature reviews Cancer.

[43] Mariani M, McHugh M, Petrillo M, Sieber S, He S, Andreoli M, Wu Z, Fiedler P, Scambia G, Shahabi S, Ferlini C (2014). HGF/c-Met axis drives cancer aggressiveness in the neo-adjuvant setting of ovarian cancer. Oncotarget.

[44] Sun C, Liu Z, Li S, Yang C, Xue R, Xi Y, Wang L, Wang S, He Q, Huang J, Xie S, Jiang W, Li D (2015). Down-regulation of c-Met and Bcl2 by microRNA-206, activates apoptosis, and inhibits tumor cell proliferation, migration and colony formation. Oncotarget.

[45] Anand S, Majeti BK, Acevedo LM, Murphy EA, Mukthavaram R, Scheppke L, Huang M, Shields DJ, Lindquist JN, Lapinski PE, King PD, Weis SM, Cheresh DA (2010). MicroRNA-132-mediated loss of p120RasGAP activates the endothelium to facilitate pathological angiogenesis. Nature medicine.

[46] Yamakuchi M, Lotterman CD, Bao C, Hruban RH, Karim B, Mendell JT, Huso D, Lowenstein CJ (2010). P53-induced microRNA-107 inhibits HIF-1 and tumor angiogenesis. Proceedings of the National Academy of Sciences of the United States of America.

[47] Wang L, Ye X, Cai X, Su J, Ma R, Yin X, Zhou X, Li H, Wang Z (2015). Curcumin suppresses cell growth and invasion and induces apoptosis by down-regulation of Skp2 pathway in glioma cells. Oncotarget.

